# The Effect of Advanced Access Implementation on Quality of Diabetes Care

**Published:** 2007-12-15

**Authors:** JoAnn M Sperl-Hillen, Leif I Solberg, Mary C Hroscikoski, A Lauren Crain, Karen I Engebretson, Patrick J O'Connor

**Affiliations:** HealthPartners Research Foundation; HealthPartners Research Foundation, Minneapolis, Minnesota; HealthPartners Research Foundation, Minneapolis, Minnesota; HealthPartners Research Foundation, Minneapolis, Minnesota; HealthPartners Research Foundation, Minneapolis, Minnesota; HealthPartners Research Foundation, Minneapolis, Minnesota

## Abstract

**Introduction:**

The study analyzes the effect of an advanced access program on quality of diabetes care.

**Methods:**

We conducted this study in a medical group of 240,000 members served by 17 primary care clinics. Seven thousand adult patients older than 18 years of age with diabetes were identified from administrative databases. Two aspects of advanced access — wait time for appointments and continuity of care — were calculated yearly for each patient during 1999 through 2001. We developed three composite measures of glucose and lipid control — process (proportion of patients with appropriate testing rates of hemoglobin A1c [HbA1c] and low-density lipoprotein [LDL]), good control (proportion with HbA1c < 8% and LDL < 130 mg/dL) and excellent control (proportion with HbA1c < 7% and LDL < 100 mg/dL) — and assessed them each year for each patient. We used multilevel logistic regression to predict the measures in 2000 and 2001 (years during and after advanced access implementation) relative to 1999 (year pre-advanced access).

**Results:**

After implementation of advanced access, wait time decreased from 21.6 days to 4.2 days, and continuity improved by 6.5% (both *P *<.01). The percentage of patients with HbA1c < 7% increased from 44.4% to 52.3% and with LDL < 100 mg/dL from 29.8% to 38.7%. Increased continuity predicted improved process (*P =* .01), good control (*P = *.033), and excellent control (*P *<.001). However, wait time did not significantly predict process (*P =* .62) or quality measures (*P =* .95).

**Conclusion:**

Measures of the quality of diabetes control improved in the year after implementation of advanced access, but better care did not correlate with decreased wait time to see a provider. However, improved continuity of care predicted improvements in both process and quality of diabetes care.

## Introduction

In 2001, the Institute of Medicine highlighted serious deficits in the quality of medical care in the United States (1). Timeliness ("reducing waits and sometimes harmful delays") was one of six domains of quality cited as having a wide gap between "the care we have and the care we could have."

To bridge this gap, many medical groups are considering ways of implementing programs to increase the availability of appointments with primary care physicians. The "open access" approach — creating walk-in clinics or holding the schedules of one or more designated physicians on a given day or week for same-day care — can decrease wait time (defined as length of wait in days until the next available appointment), but it does not necessarily improve continuity of care because patients often see doctors other than their own (2,3). Another approach — advanced access (4,5) — encourages providers to see their own patients on the day a patient requests a visit, thus improving continuity of care (6). The benefits of advanced access on appointment availability have been reported (7).

We aimed to determine whether implementation of advanced access affected composite measures of diabetes care. Specifically, we explored the effect of the improved availability of appointments and continuity resulting from advanced access on quality measures of care for patients with diabetes. Ours is the first report of the effect of advanced access on the quality of care of patients with a chronic disease.

Our study population comprised 7000 diabetes patients in a large medical group owned by a health plan. Improvement in care of patients with diabetes had been a priority of the medical group since about 1995 (8). In late 1999, medical group leaders decided to undertake a major intervention to increase the availability of appointments, thus increasing patient satisfaction; overall efficiency; and, ultimately, plan membership. The advanced access model was selected, and the intervention began in 2000. Consultants engaged to help with the change conducted a series of 1- to 2-day workshops during 2000 for improvement teams from all 17 of the group's primary care clinics. Consultants provided training, and a care improvement team provided regular measurement and feedback of appointment availability to providers and clinics. Advertisements in the media informed patients about the better availability of clinic appointments with their regular physicians. Elimination of more than 100 appointment types with different stipulations based on provider preferences and transformation to standardized appointment types enabled scheduling of most appointments for the same amount of time, regardless of visit reason. The intervention attempted to eliminate frozen slots (schedule holds [i.e., time on a provider's regular work schedule not made available for scheduled patients and often used for paper work and phone calls]) and required physicians to work extra hours to see patients who already had been scheduled for future appointments. Because patients received no systematic reminder calls for recommended follow-up appointments before or after advanced access implementation, change in continuity measure could not be attributed to reminder mechanisms.

## Methods

We conducted this study in a 550-physician multispecialty medical group that has 17 primary care clinics and is owned by a health plan. The plan comprises 650,000 members, of whom 240,000 are cared for by this medical group. We identified 7000 adult patients (older than 18 years of age) with diabetes from health plan administrative databases using algorithms modified from a previously described approach and validated against chart audits (9). Patients included in the study had to be enrolled in the plan for 11 of 12 months of each study year and to have two or more specific *International Classification of Diseases, Ninth Revision*, outpatient diabetes diagnostic codes, one inpatient diagnostic code, or a filled prescription for diabetes-specific medication within a 1-year period. We excluded patients with gestational diabetes. We also excluded patients taking metformin alone who did not have a diabetes code because chart audits indicated that providers used metformin to treat impaired glucose tolerance and polycystic ovary disease as well as to treat diabetes. Chart audits demonstrated a positive predictive value of 0.97 for this diabetes identification method.

We identified patients who met these criteria for 1999 through 2001, and included each patient in the analysis for each year he or she was identified as having diabetes. We analyzed serial cross-sections of patients with diabetes to assess whether systematic changes in access were related to improvements in care for all patients with diabetes, not just those who were continuously enrolled.

We developed three composite measures of glucose and lipid control — process, good control, and excellent control — and assessed them each year for each patient. *Process* assessed whether each patient had two or more hemoglobin A1c (HbA1c) tests and one or more fasting lipid profile tests during the calendar year. For patients with at least one HbA1c and one low-density lipoprotein (LDL) test in a calendar year, two yearly composite outcome measures were assessed: *good diabetes control* and *excellent diabetes control*. We defined good control as both HbA1c < 8% and LDL < 130 mg/dL and excellent control as both HbA1c < 7% and LDL < 100 mg/dL.

We calculated two aspects of advanced access — wait time for appointments and continuity of care — yearly for each patient. We measured wait time using the commonly accepted measure (10) of days to third next available appointment with a provider in the patient's primary clinic and monitored the improvement with advanced access implementation using existing administrative data collected at each clinic. We calculated continuity of care on the basis of visits by a patient to different providers during each year (11). The formula is [Σ(visit_i_^2^) − Σvisit]/[Σvisit ×(Σvisit − 1)] (where i = specific providers), yielding a number ranging from 0 to 1 (where higher numbers indicate more continuity of care with a single provider) (12).

We calculated age, sex, and known coronary artery disease (CAD) yearly for each patient and as covariates in the analysis. We included CAD as a covariate because of the differential lipid goals for patients with known CAD and because it is the most common cause of morbidity and mortality for people with diabetes. We also included the mean number of outpatient, primary care, and urgent care visits by these patients in each of the three study years.

We used multilevel logistic regression to predict performance for the three composite measures in 2000 and 2001 relative to 1999, controlling for age, sex, and CAD. A second set of analyses predicted the composite measures from the continuous wait time and continuity measures separately, controlling for age, sex, CAD, and year. Finally, a third set of analyses predicted the composite measures from yearly measured provider access and continuity of care, controlling for age, sex, CAD, and year, so the relation between each component of the advanced access model used by this medical group and the composite measures could be estimated while controlling for the other. Approximate *P* values for fixed parameters were estimated from the parameter coefficients and their standard errors. A three-level (patient-year within patient within provider) model was specified for all analyses so the analysis included multiple yearly observations per person and controlled for the multiple correlated observations within patients (i.e., multiple observations per patient) and within providers (i.e., multiple patients within provider) structure.

The HealthPartners Institutional Review Board reviewed, approved in advance, and monitored all steps in the development of the identification system, aggregation of data, and data analysis. Because the analysis used aggregate de-identified claims data, the institutional review board did not require informed consent.

## Results

During the baseline year (1999), 44.4% of diabetes patients had an HbA1c < 7%, and 29.8% had an LDL < 100 mg/dL, improving to 52.7% and 38.7%, respectively, in 2001 (Table 1). Both wait time and continuity significantly improved during the study period. Process improved in 2000 compared with 1999, and both good control and excellent control improved during 2000 and 2001 compared with 1999.

Continuity of care was significantly related to each of the three composite measures (Table 2). Higher continuity scores were associated with a higher predicted proportion of patients meeting each measure. Wait time was not significantly related to any of the three measures.

Finally, the third set of analyses predicted the composite measures from a model that included wait time and continuity simultaneously. With both of these advanced access characteristics in the model, greater continuity of care continued to predict a higher proportion of patients meeting the process (*P *<.001) and excellent control (*P =* .017) measures. Prediction for good control no longer was statistically significant. This final set of analyses demonstrated that continuity rather than shorter wait time was associated with improvement in care.

## Discussion

In our study, appointment availability increased and continuity of care improved after implementation of advanced access in a large patient population. However, only continuity of care was associated with improved care. Other benefits of improving continuity of care, which have been well documented, include more positive visit experiences by patients (13) and reduced use of resources — and thus reduced costs (14). A recent study using evidence from the Third National Health and Nutrition Examination Survey (NHANES) concluded that good glucose control is more likely among people with diabetes who regularly see their usual physician or health provider to manage their condition (15). Our study observations are consistent with previous studies of continuity of care for patients with chronic diseases (16-18) and support the hypothesis that quality of diabetes care could be improved by improving continuity of care by primary care physicians.

Although advanced access is proposed to improve continuity of care, theoretically by reducing wait time to see a provider, we did not find a direct relation between wait time and improved care. Additional analyses showed that shorter provider wait time was only weakly associated with increased continuity of care (*P =* .02). Therefore, gains in continuity of care should be attributed only cautiously to advanced access.

Several other changes in the medical group's care system made just before implementation of advanced access to support the change also might have substantially affected continuity of care. First, payment of physicians changed from a fixed yearly salary to a productivity-based provider reimbursement, leading to a general increase in physician productivity. In addition, centralized scheduling (19) facilitated standardized appointment types and scheduling efficiency (lower costs), which also could improve continuity. Thus, our findings that suggest relations among these variables (advanced access, wait time, and continuity) might be more complex than generally believed and might suggest an important direction for future research.

Composite measures of process and quality reflect the importance of managing both risk for CAD and glucose control in patients with diabetes. Although individual measures of HbA1c and LDL improved after advanced access implementation, we analyzed composite measures of process and quality because they are considered more representative of overall change in the system of care and have become the recommended measurement for diabetes in Minnesota (20). This methodology was highlighted recently in the Institute of Medicine report on national health care measurement standards (21). The results might look unimpressive, but small improvements in composite measurement represent simultaneous improvements in multiple clinical domains and possibly gains in care quality.

When our model incorporated both wait time and continuity, continuity was linked to the composite measure of excellent control but not of good control. This discrepancy might be explained in part by the increasing importance of continuity of care as care goals become more ambitious and presumably more difficult for patients to achieve. Recent national guidelines for diabetes have lowered the recommended levels of HbA1c and LDL beyond even the measures of excellent control used in this study (22,23), possibly increasing the importance of continuity of care.

Self-reported continuity of care is strongly associated with higher patient satisfaction (24). Yearly satisfaction surveys of a sample of the patients in our study after advanced access implementation showed significant increases — from 36% to 55% — in patients reporting being very satisfied. Although the manner in which care is delivered most likely influences satisfaction, satisfaction cannot be attributed directly to either decreased wait time or continuity of care.

Some physicians have suggested that access models best serve patients with acute problems at the expense of patients with chronic diseases (7,19). Implementation of advanced access in this medical group met with physician resistance because physicians were encouraged to discontinue ingrained scheduling practices, such as limits on the number of double bookings, longer appointments for patients with chronic diseases or complaints thought to require more visit time, and use of frozen slots as catch-up time. Many physicians worked overtime to reduce the backlog of patients already scheduled well into the future. Physicians questioned how advanced access would affect quality of care (19). Our results show that diabetes care and patient satisfaction improved after successful implementation of advanced access in primary care in this medical group. Our study should temper criticism of advanced access as an "acute care model" and provider concerns about deleterious effects on patients with chronic disease. At the very least, shorter wait time for appointments did not negatively affect glucose and lipid control in patients with diabetes. Moreover, if the approach to improving appointment availability strongly emphasizes improving both continuity of care and access time, the impact may be positive.

The results of our study are subject to several limitations. First, because this study was conducted at one multiclinic medical group, our results might not be generalizable to other settings, particularly if the baseline characteristics (appointment availability and continuity) differ substantially. Future studies of models to improve appointment access should consider the specifics of the access model being evaluated (e.g., advanced access, open access); some models promote physician-level continuity of care, whereas others might not. Second, our study lacked a control group and might not have accounted for secular trends. NHANES data showed that glucose control rates (defined as the proportion of patients with HbA1c < 7%) declined from 1994 to 2000 (25). Therefore, secular trends over the study period are unlikely to explain the study results. Third, diabetes measures in the community where this study took place have been better than national measures (26) and could be attributed to multiple initiatives other than advanced access implementation alone. We addressed the concern about historical trend by including year of observation in the regression models predicting the composite care measures from wait time and continuity of care; however, including year in these models also could have diminished the effect of wait time on diabetes care if no other strong historical trends accounted for change during the study period.

Despite these limitations, our quantitative study is the first to compare the effects of change in access and continuity of care on the quality of care for patients with diabetes. The type of advanced access program implemented in this medical group, which promoted both continuity of care and same-day access for appointments, resulted in improved glucose and lipid control in adults with diabetes. The results suggest that reduced wait time does not deleteriously affect diabetes care. The study also reinforces the results of previous studies showing an association between improved continuity of primary care and better diabetes control.

## Figures and Tables

**Table 1:** Characteristics of Patients With Diabetes Before (1999), During (2000), and After (2001) Implementation of Advanced Access Program, 1999–2001

Characteristic	1999	2000	2000 vs 1999	2001	2001 vs 1999

	N = 6741	N = 7056	*P*	N = 7238	*P*
Age in 1998, y	59.7	59.1	.01	58.1	<.001
Sex, % male	54.0%	53.2%	.32	53.0%	.19
Coronary artery disease	14.3%	15.5%	.06	15.6%	.04
Wait time, d^a^	21.6	11.0	<.001	4.2	<.001
Continuity of care^a^,^b^	.681	.699	.001	.725	<.001
≥2 HbA1c tests^a^	4.5%	66.0%	.01	60.6%	<.001
≥1 LDL test^a^	61.2%	68.7%	<.001	67.9%	<.001
HbA1c < 7%^a^	44.4%	48.7%	<.001	52.3%	<.001
HbA1c < 8%^a^	69.5%	73.5%	<.001	76.5%	<.001
LDL < 100 mg/dL^a^	29.8%	35.1%	<.001	38.7%	<.001
LDL < 130 mg/dL^a^	65.1%	69.6%	<.001	71.8%	<.001
Process^a^	46.9%	52.4%	<.001	48.8%	.11
Good control^a^,^c^	48.7%	54.2%	<.001	58.1%	<.001
Excellent control^a^,^d^	14.6%	18.3%	<.001	21.8%	<.001
No. of primary care visits	3.89	4.56	<.001	4.49	<.001
No. of diabetes-related primary care visits	2.37	2.73	<.001	2.50	<.001
≥1 Urgent care visit or emergency department visit	41.0%	40.1%	.26	37.6%	<.001

HbA1c indicates hemoglobin A1c; LDL, low-density lipoprotein.

a Comparison controlled for age, sex, and coronary artery disease.

b Continuity of care = proportion of visits with a single provider.

c Good control defined as both HbA1c < 8% and LDL < 130 mg/dL.

d Excellent control, as both HbA1c < 7% and LDL < 100 mg/dL.

**Table 2:** Model-Based Predicted Proportion of Patients Meeting Three Measures of Glucose and Lipid Control According to Levels of Continuity of Care and Appointment Wait Time, Advanced Access Program, 1999–2001^a^

Measure^b^	Continuity Score^c^				* P*

	0.25	0.50	0.75	1.0	
Process	47.3	48.0	48.8	49.6	.01
Good control	38.5	39.3	40.1	40.9	.03
Excellent control	3.7	3.9	4.2	4.5	<.001

**Measure^b^**	**Wait Time Percentile^d^**				*** P ***

	12.5	37.5	62.5	87.5	
Process	46.8	46.7	46.6	46.2	.62
Good control	39.6	39.6	39.6	39.7	.95
Excellent control	4.2	4.2	4.2	4.2	.98

a All values are percentages.

b Multilevel logistic regression controlled for age, sex, coronary artery disease, and year.

c A score ranging from 0 to 1, where high numbers indicate a greater proportion of visits with a single provider.

d Length of wait in days until the next available appointment. A precise number of days' wait depends on the setting (e.g., 5 days might be considered a short wait in some settings but long in others). For this reason, wait time divided into quartiles and expressed as the median percentile for each quartile (median of 0-25th percentile is 12.5). Wait time percentile should be interpreted such that a low percentile corresponds to less access (more days), whereas a higher percentile corresponds to more access (fewer days).
